# Effect of Attenuation of Treg during BCG Immunization on Anti-Mycobacterial Th1 Responses and Protection against *Mycobacterium tuberculosis*

**DOI:** 10.1371/journal.pone.0002833

**Published:** 2008-07-30

**Authors:** Barbara Jaron, Eddie Maranghi, Claude Leclerc, Laleh Majlessi

**Affiliations:** 1 Unité de Régulation Immunitaire et Vaccinologie, Institut Pasteur, Paris, France; 2 Animalerie Centrale, Institut Pasteur, Paris, France; 3 Institut National de la Santé et de la Recherche Médicale U883, Paris, France; New York University School of Medicine, United States of America

## Abstract

**Background:**

The functional equilibrium between natural regulatory T cells (Treg) and effector T cells can affect the issue of numerous infections. In unvaccinated mice, the influence of Treg in the control of primary infection with mycobacteria remains controversial.

**Methodology:**

Here, we evaluated the role of Treg during prophylactic vaccination with *Mycobacterium bovis* BCG (Bacillus Calmette-Guérin) on the induction of T cell responses and on the protective effect against subsequent *M. tuberculosis* challenge in mice.

**Principal Findings:**

We demonstrated that, subsequent to BCG injection, Treg were recruited to the draining lymph nodes and negatively control anti-mycobacterial CD4^+^ — but not CD8^+^ — T-cell responses. Treatment of BCG-immunized mice with an anti-CD25 mAb (PC61) induced an increase IFN-γ response against both subdominant and immunodominant regions of the protective immunogen TB10.4. In Treg-attenuated, BCG-immunized mice, which were then infected with *M. tuberculosis*, the lung mycobacterial load was significantly, albeit moderately, reduced compared to the control mice.

**Conclusions:**

Our results provide the first demonstration that attenuation of Treg subset concomitant to BCG vaccination has a positive, yet limited, impact on the protective capacity of this vaccine against infection with *M. tuberculosis*. Thus, for rational design of improved BCG, it should be considered that, although the action of Treg does not represent the major cause of the limited efficiency of BCG, the impact of this cell population on the subsequent control of *M. tuberculosis* growth is significant and measurable.

## Introduction

The only vaccine available against infection with *Mycobacterium tuberculosis* is the live attenuated *M. bovis* BCG (Bacillus Calmette-Guérin). With more than three billion of doses injected, BCG is the most used vaccine in the world, although its efficiency is highly variable. Even though, in non-endemic zones, BCG is efficient up to 80% against disseminated infections with *M. tuberculosis*, this vaccine is not able to protect efficiently against the adult pulmonary tuberculosis in endemic zones. Moreover, the resurgence of tuberculosis in immuno-compromised individuals and the rapid expansion of multi-drug resistant and extensively drug resistant tuberculosis [1] reinforce the need of fundamental understanding of the variability and the limited efficiency of BCG for a better rational design of new strategies of anti-tuberculosis vaccines [2].

Variability in the efficiency of BCG against infection with *M. tuberculosis* can be explained by differences in vaccination protocols and in BCG strains used in different areas of the world and human genetic factors, predisposing individuals to develop an active tuberculosis [3], [4]. Moreover, this variability can also be due, in endemic zones, to pre-exposure to environmental mycobacteria, such as *M. avium*, which could induce immune responses against antigens shared with BCG. The resulted immune cross-recognition may decrease the persistence of subsequently injected BCG and may lead to variable protective efficiency [5], [6]. On the other hand, the limited efficiency of BCG against infection with *M. tuberculosis* can be explained by the following hypotheses. (i) Deletion of genes coding for protective immunogens in BCG and/or over attenuation of BCG, due to the loss of genetic Regions of Differences (RD) 7, 8 could explain the weak efficiency of BCG. This hypothesis has been confirmed by genetic reintroduction of RD1 into the BCG Pasteur strain [9], [10]. The resulted BCG::RD1 is able to induce marked Th1 responses to *M. tuberculosis*-specific antigens, i.e., Early Secreted Antigenic Target, 6 kDa (ESAT-6^3^) and Culture Filtrate Protein, 10 kDa. It displays an increased virulence and persistence, which are in direct correlation with the increased protective capacity of this vaccine candidate against *M. tuberculosis* [10], [11] (ii) Despite the induction of anti-mycobacterial Th1 cells by BCG immunization, intracellular mycobacteria sequestered within granuloma, may be inaccessible to these effector T cells. (iii) Finally, subsequent to BCG immunization, concomitant induction of regulatory T cells (Treg) [12], in parallel to the induction of anti-mycobacterial T-cell responses, could reduce the efficiency of the latter in protection against *M. tuberculosis*.

During infection, like IL10 and TGF-β, the principal function of CD4^+^ CD25^+^ FoxP3^+^ Treg is the control of intense inflammation, induced at the site of infection, to avoid tissue damage. However, this immune regulation can counteract the function of CD4^+^ or CD8^+^ effector T cells and paradoxically dampen the control of infection. Subsequent to the pioneering work on the role of Treg in the persistence of *Leishmania major* in mice [13], it has been shown that the functional balance between natural Treg and effector T cells can influence the control of numerous viral, bacterial or parasitic infections [14].

With respect to the control of primary mycobacterial infections in unvaccinated mice, the role of Treg remains controversial in the few works addressing this question. Indeed, Quinn *et al.* showed that anti-CD25 mAb treatment in mice has no consequence on the control of mycobacterial growth [15]. In contrast, when effector T cells and Treg were adoptively co-transferred, at high Treg/effector T cells ratio into recipients, Treg were able to significantly inhibit the action of effector T cells on the control of *M. tuberculosis* growth [16]. An improved control of *M. tuberculosis* infection was also observed under conditions of total depletion of FoxP3^+^ T cells, giving rise to a substantial polyclonal — but not mycobacteria-specific — activation of T cells [17]. Besides these discrepancies, it is important to know whether the prophylactic anti-mycobacterial vaccination with BCG leads to induction of Treg, with the potential to influence the T-cell mediated control of subsequent infection with virulent *M. tuberculosis*. Here, by use of a standard mouse model of anti-tuberculosis vaccine assay [18], we studied the possible recruitment/expansion of Treg in mice immunized s.c. with BCG or infected with *M. tuberculosis* via aerosol route. By modulating the Treg compartment during BCG immunization, we evaluated the role of these cells on the set up of anti-mycobacterial CD4^+^ or CD8^+^ T-cell responses. Finally, in mice immunized with BCG and treated with anti-CD25 mAb (PC61), we investigated the T-cell responses at the site of infection and the outcome of *M. tuberculosis* infection. Our results show that, during immunization with BCG, attenuation of Treg function by anti-CD25 mAb (PC61) treatment, finely tunes anti-mycobacterial Th1 responses and slightly, yet significantly, improves the protective efficiency of BCG against infection with *M. tuberculosis*.

## Results

### Recruitment/expansion of Treg in lymphoid organs of BCG-immunized mice

**Figure 1:**
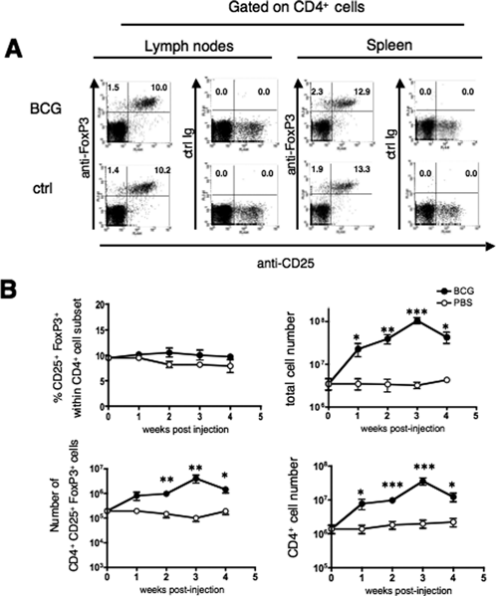
Analysis of Treg subset in lymphoid organs of BCG immunized mice. (A) Percentages of CD25^+^ FoxP3^+^ cells within the CD4^+^ T-cell subset in inguinal lymph nodes or spleen of BALB/c mice, untreated or immunized s.c. with 1×10^6^ CFU of BCG, at three weeks post-immunization. Results are representative of two independent experiments. Three mice were analyzed individually and one mouse out of three is depicted. The numbers indicated in the windows are percentages of the corresponding cells. (B) Kinetics of recruitment/expansion of CD25^+^ FoxP3^+^ T cells in draining lymph nodes of BCG-immunized mice. Data are percentages of CD25^+^ FoxP3^+^ cells within the CD4^+^ T-cell subset (left, top), number of CD4^+^ CD25^+^ FoxP3^+^ cells (left, bottom), total cell number (right, top) or number of CD4^+^ T cells (right, bottom), in the inguinal lymph nodes of PBS-injected or BCG-immunized mice. Results are means±SD of three mice, studied individually. *, ** or *** = statistically significant, as determined by Student's *t* test, *p*<0.05, 0.02 or 0.01, respectively.

We first analyzed the possible recruitment/expansion of Treg in the inguinal draining lymph nodes and spleen of BALB/c mice immunized s.c. with 1×10^6^ CFU of BCG. At 3 weeks post-immunization, percentages of CD25^+^ FoxP3^+^, and also of CD25^−^ FoxP3^+^, within the CD4^+^ T-cell compartment of the inguinal lymph nodes and of the spleen of BCG-immunized mice (Figure 1A, top) were comparable to those of age-matched non-immunized controls (Figure 1A, bottom). However, after s.c. immunization with BCG, following a 4-week-kinetics study, we observed that, in the inguinal lymph nodes, despite the non variable percentage of CD25^+^ FoxP3^+^ within the CD4^+^ T-cell subset (Figure 1B top, left), the absolute number of these cells statistically increased (Figure 1B bottom, left), albeit in a comparable manner to the increase of the number of total lymph node cells (Figure 1B top, right), or of the number of CD4^+^ T cells (Figure 1B bottom, right). The increased Treg numbers in the draining lymph nodes may result from their recruitment, local proliferation/retention or conversion from CD4^+^ CD25^−^ T cells. No change was detected in the number of CD4^+^ CD25^+^ FoxP3^+^ cells in the spleen (data not shown). Thus, the size of the Treg compartment grows proportionally to that of the total cells and of the CD4^+^ T-cell subset in the draining lymph nodes of BCG-immunized mice.

### Treg-mediated negative regulation of anti-mycobacterial CD4^+^ T-cell responses in BCG-immunized mice

**Figure 2:**
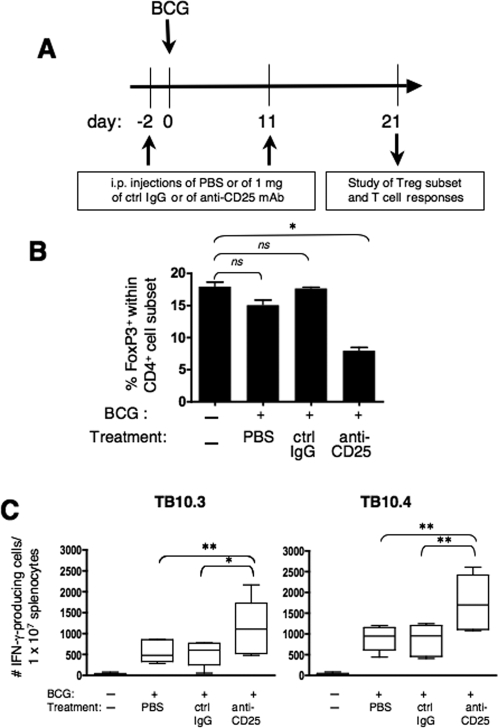
Effect of anti-CD25 mAb treatment on Treg compartment and on anti-mycobacterial Th1 responses. (A) Mice received, at days −2 and 11, i.p. injections of PBS or of 1 mg of the control rat IgG (CRL-1912) or of anti-CD25 mAb (PC61). At day 0, mice were immunized s.c. with 1×10^6^ CFU of BCG. (B) At day 21, percentages of FoxP3^+^ cells within CD4^+^ T cell splenocytes were determined. Results represent mean±SD obtained in individually studied mice (*n* = 3). * = statistically significant, as determined by Anova test, *p*<0.001. (C) Frequency of IFN-γ producing T splenocytes in response to TB10.3 or TB10.4 in mice immunized by BCG and treated with anti-CD25 mAb. At day 21 post-immunization, serial dilutions of splenocytes from mice (*n* = 6), unvaccinated or immunized with BCG and injected with PBS, control IgG or PC61 mAb, as described in (A), were stimulated *in vitro* with 10 µg/ml of TB10.3:74–88 (left) or TB10.4:74–88 (right) to determine the frequency of specific CD4^+^ IFN-γ-producing T cells by ELISPOT. Horizontal bars and the boxes indicate the mean values and SD, respectively. Whiskers show the smallest and the largest values. * or ** = statistically significant, as determined by Student's *t* test, *p*<0.02 or 0.01, respectively. *Ns* = no significant difference.

To investigate the possible role of Treg on anti-mycobacterial T-cell responses following BCG immunization, we evaluated IFN-γ CD4^+^ T-cell responses of mice immunized by BCG and treated with anti-CD25 mAb (PC61). As shown in the Figure 2A, mice received i.p., at day −2 and day 11, PBS or 1 mg of a control rat IgG or PC61. At day 0, mice were immunized s.c. with 1×10^6^ CFU of BCG. At day 15 (not shown), as well as at day 21 (Figure 2B), we observed a significant decrease (Anova, *p*<0.001) in the percentage of FoxP3^+^ within CD4^+^ T splenocytes of anti-CD25-treated mice, compared to their counterparts injected with PBS or with the control IgG (Figure 2B). Compared to BCG-vaccinated individuals, unvaccinated mice, treated with PC61 displayed the same range of reduction in percentages of FoxP3^+^ within the CD4^+^ cell subset (not shown). The partial depletion of CD4^+^ FoxP3^+^ T cells in anti-CD25 mAb-treated mice has been previously described [19]–[21]. It is noteworthy that such anti-CD25 mAb treatment with PC61 does allow efficient attenuation of the Treg function in numerous studies and in different models. Moreover, this anti-CD25 mAb treatment does not block the action of activated T cells, as it is reported in many cases, T-cell responses increased following this treatment [19], [22]–[24].

At day 21, we studied IFN-γ CD4^+^ T-cell responses of these mice against TB10.3 (Rv3019c) and TB10.4 (Rv0288) mycobacterial immunogens. TB10.3 and TB10.4 are expressed by both BCG and *M. tuberculosis* and belong to the immunogenic family of ESAT-6 proteins [25]. Moreover, TB10.4 has been shown to be a protective immunogen [26], [27]. The frequencies of IFN-γ-producing CD4^+^ T splenocytes, specific to MHC-II-restricted immunodominant epitopes contained in TB10.3:74–88 or TB10.4:74–88 peptides (Figure 2C), were significantly increased in anti-CD25-treated mice, compared to the control mice injected with PBS or with the control IgG. IFN-γ responses to these peptides were only due to CD4^+^ T cells, as shown by CD4^+^ T-cell depletion that fully abolished these responses [26]. It is noteworthy that in anti-CD25 mAb-treated mice, the partial depletion of Treg corresponds to a loss of 2 to 2.5% of total splenocytes and thus cannot account for the observed increase in the frequency of IFN-γ-producing splenocytes. Stimulation with the negative control peptide PV1:103–116 did not reveal any IFN-γ producing cells (not shown).

**Figure 3:**
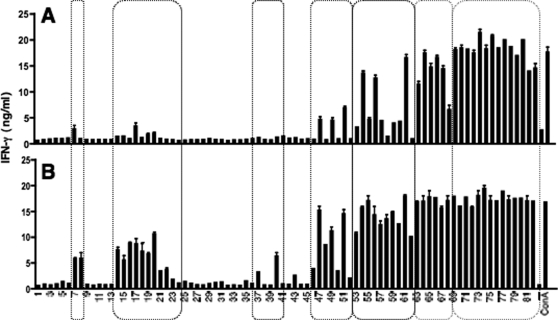
Effect of anti-CD25 mAb treatment on Th1 responses to immunodominant or subdominant regions of TB10.4 immunogen. Effect of anti-CD25 mAb treatment on Treg anti-mycobacterial Th1 responses to 15-mer overlapping peptides covering the entire sequence of TB10.4. Mice were treated with the control rat IgG (CRL-1912) (A) or with anti-CD25 mAb (PC61) (B) as described in Figure 2A. At day 21, splenocytes from individual mice were stimulated *in vitro* with 20 µg/ml of individual 15-mer overlapping peptides covering the entire sequence of TB10.4. IFN-γ secretion in the culture supernatant was assessed by ELISA at 72 h. Three mice were individually studied per experimental group and one representative individual is shown.

Based on the fact that responses to all T-cell epitopes are not affected equally by Treg, it has been suggested that these cells could modify the profile of T-cell responses to immunodominant or subdominant regions [28]. We thus sought to determine whether, under the conditions of Treg attenuation, the profile of Th1 responses to immunodominant and subdominant regions of a protective mycobacterial immunogen is modified. To this end, we evaluated, in individual BCG-immunized mice (*n* = 3), treated with the control IgG or anti-CD25 (PC61) mAb, as detailed in Figure 2A, IFN-γ production by splenocytes stimulated with individual overlapping 15-mer peptides, covering the entire sequence of TB10.4 at a single amino acid step [26]. Unvaccinated control mice did not produce IFN-γ after *in vitro* stimulation with these peptides (data not shown, [26]). We previously established that IFN-γ responses detected in these conditions with TB10.4 15-mer peptides are only due to the CD4^+^ T-cell subset [26]. As shown in Figure 3, TB10.4:7–22 (peptides 7 and 8), TB10.4:14–38 (peptides 14 to 24), TB10.4:37–51 (peptide 37) and TB10.4:40–54 (peptide 40), were very slightly immunogenic in control IgG-injected mice (Figure 3A). However, these peptides triggered more intense responses in PC61-treated mice (Figure 3B).

The immunogenic TB10.4:46–65 (peptides 46–51), TB10.4:53–75 (peptides 53–61) and TB10.4:63–82 (peptides 63–68) regions triggered detectable responses in control IgG-treated mice (Figure 3A), but stimulate higher responses in PC61-treated mice (Figure 3B). In this pepscan assay, no differences were detectable between the two experimental groups with respect to the immunogenicity of the C-terminal TB10.4:69–96 (peptides 69–82) region. This could be due to the high peptide concentration (20 µg/ml) used in the pepscan assay to detect responses against weak epitopes. Indeed, compared to their IgG-treated counterparts, in the PC61-treated, BCG-immunized mice, higher IFN-γ responses to TB10.4:74–88 were detected by use of ELISPOT (Figure 2C) or by ELISA in the supernatant of cultures stimulated with TB10.4:74–88 concentrations, ranging from 0.1 to 100 ng/ml (not shown). Altogether, these results show that Th1 responses to both immunodominant and subdominant regions of TB10.4 protective immunogen are under the negative control of Treg during BCG immunization.

### Absence of Treg-mediated negative regulation of anti-mycobacterial CD8^+^ T-cell responses in BCG-immunized mice

**Figure 4:**
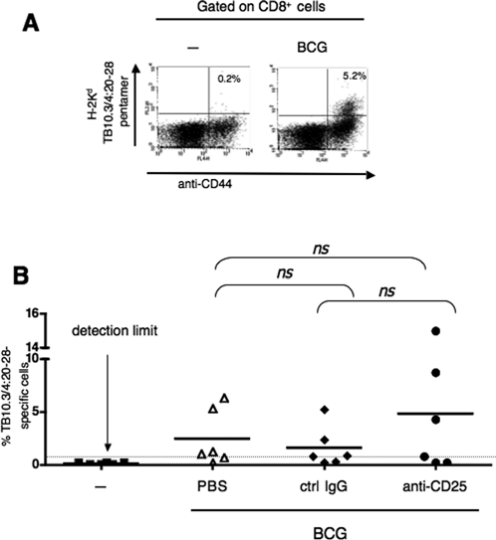
CD8^+^ T-cell responses in anti-CD25 mAb treated, BCG-immunized mice. (A) Representative CD8^+^ T-cell response to TB10.3 or TB10.4 in BALB/c mice immunized by BCG compared to control non-immunized mice. At day 21 post-immunization, splenocytes were stimulated *in vitro* with 10 µg/ml of TB10.3/4:20–28, containing a H-2K^d^-restricted immunodominant epitope. Six days later, splenocytes were stained with a combination of FITC-anti-CD8, allophycocyanine-anti-CD44 and PE-conjugated H-2K^d^ pentamer complexed with TB10.3/4:20–28 peptide. Cells were then analyzed by cytofluorometry. (B) Comparison of CD8^+^ T-cell response to TB10.3 or TB10.4 in individual mice, injected with PBS, the control IgG or PC61 mAb and immunized with BCG, as described in the Figure 2. Percentages of pentamer^+^ CD44^+^ cells within the CD8^+^ T-cell population are shown in individual mice. Results are pooled from two independent experiments. *Ns*, no significant difference as determined by Student's *t* test.

Based on the demonstration that Treg are able to down-regulate CD8^+^ T cell responses in immunization against *Listeria monocytogenes* in mouse model [29], we evaluated CD8^+^ T-cell responses of BCG-immunized BALB/c mice, injected with PBS, the control IgG or anti-CD25 mAb, as shown in the Figure 2A. At day 21, splenocytes from individual mice were stimulated with TB10.3/4:20–28 peptide, containing a MHC-I-restricted epitope. The frequency of specific T cells within CD8^+^ T-cell population was determined, as shown in Figure 4A, by use of a combination of anti-CD8, anti-CD44 mAbs and a H-2K^d^ pentamer complexed with the homologous peptide [30], [31]. We observed that, compared to the control groups, the anti-CD25 treatment of BCG-immunized mice, slightly but not significantly, increased the percentage of TB10.3/4:20–28-specific CD8^+^ T cells in the responding mice and did not improve the frequency of mice with detectable TB10.3/4:20–28-specific CD8^+^ T cells (Figure 4B). Accordingly, in the numerous experiments that we performed to evaluate anti-mycobacterial CD8^+^ T-cell responses specific to Ag85A or TB10.3/4, using either the ^51^Cr release cytotoxicity assay or the MHC-I pentamer FACS assay, we always detected only 30 to 50% of positive mice in BCG-immunized groups with large variability in percentages of anti-mycobacterial CD8^+^ T cells among individuals of the same experimental group [30, data not shown].

### Recruitment/expansion of Treg in lung parenchyma of *M. tuberculosis*-infected mice

**Figure 5:**
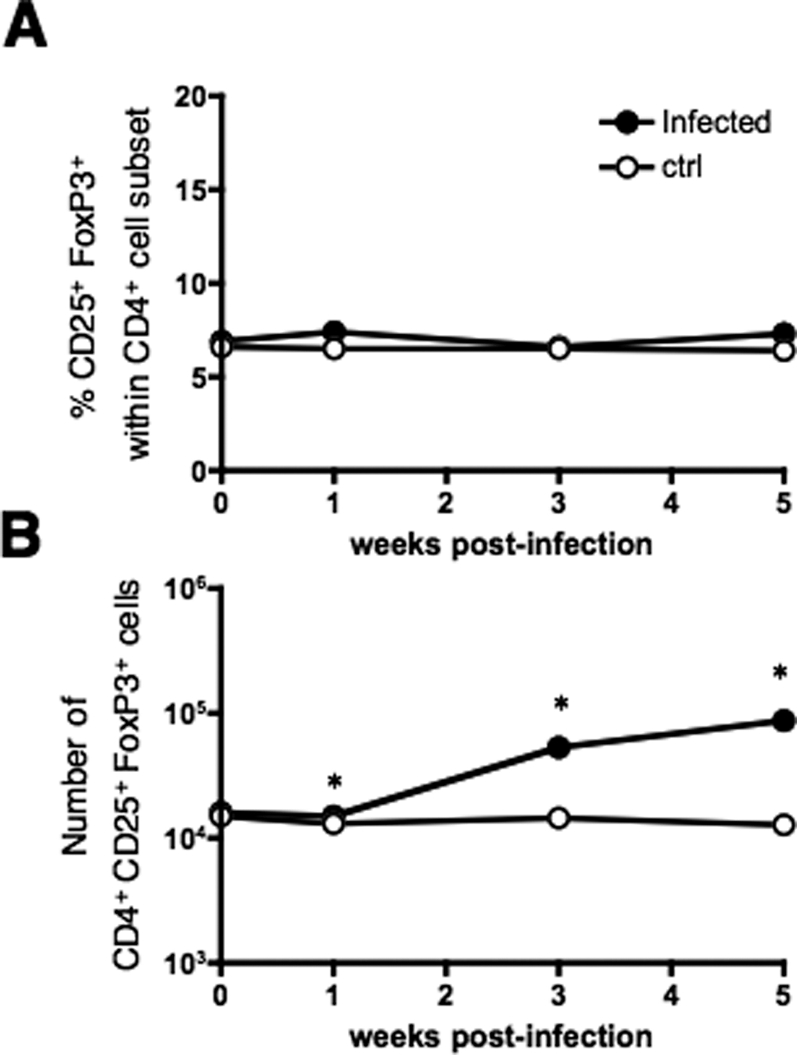
Analysis of CD25^+^ FoxP3^+^ T cells in the lung parenchyma of *M. tuberculosis*-infected mice. (A) Percentages of CD25^+^ FoxP3^+^ cells within the CD4^+^ T-cell subset in lung parenchyma of untreated controls or of mice infected with ≈100 CFU of H37Rv by aerosol route and (B) number of CD4^+^ CD25^+^ FoxP3^+^ cells, in the lung parenchyma of control or *M. tuberculosis*-infected mice. Results are means±SD of three mice, studied individually. *, statistically significant (Student's *t* test, *p*<0.02).

To investigate the recruitment/expansion of Treg subset at the site of *M. tuberculosis* infection, we infected BALB/c mice with ≈100 CFU of *M. tuberculosis* H37Rv by aerosol route. Similar to the draining lymph nodes of BCG-immunized mice, following a 4-week-kinetics study of the lung parenchyma, despite the constant percentage of CD25^+^ FoxP3^+^ cells (Figure 5A), and CD25^−^ FoxP3^+^ within the CD4^+^ T-cell subset the absolute number of these cells statistically increased in the infected mice (Figure 5B), in parallel to the well-characterized increase in the total number of lymphocytes or of CD4^+^ T cells recovered from the infected lungs [32].

### Study of Th1 responses in anti-CD25-treated, BCG-immunized and *M. tuberculosis*-infected mice

**Figure 6:**
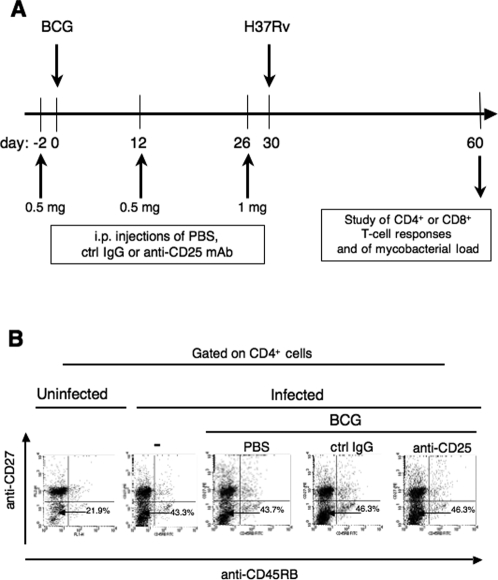
Effect of anti-CD25 mAb treatment on CD4^+^ T cells of BCG-immunized, *M. tuberculosis*-infected mice. (A) Protocol of i.p. anti-CD25 (PC61) mAb injections, s.c. BCG immunization, aerosol *M. tuberculosis* challenge and of study of T cell functions and mycobacterial load in the lung parenchyma of BALB/c mice. (B) Activated/effector CD4^+^ T cells in the lung parenchyma of these mice. At one month post-challenge, pooled lymphocytes from lung parenchyma of each experimental group were stained with a combination of allophycocyanine-anti-CD4, FITC-anti-CD45RB and PE-anti-CD27 mAbs and analyzed by cytofluorometry.

We then wondered whether, in the conditions of BCG vaccination, Treg exert any inhibitory effect on the induction or on the action of T cell-responses, responsible of the partial control of subsequent *M. tuberculosis* challenge. To address this issue BALB/c mice received, at days −2, 12 and 26, i.p. injections of PBS, of control IgG or of anti-CD25 mAb (Figure 6A). At day 0, mice were immunized s.c. with 1×10^6^ CFU of BCG. At day 30, all groups were challenged by ≈ 100 CFU/mouse of *M. tuberculosis* H37Rv by aerosol route.

We analyzed anti-mycobacterial T-cell responses in these experimental groups. Antigen-experienced CD4^+^ T cells accumulating within mycobacteria-infected lung parenchyma can be divided into CD27^+^ and CD27^−^ subsets. Only the CD27^−^ population contains highly differentiated, IFN-γ-producing T cells [33]. Thus, determination of percentages of activated CD27^−^ cells represents an appropriate way to measure Th1 response at the site of mycobacterial infection. At one month post-challenge, we detected a markedly increased percentage of CD45RB^−^ CD27^−^ cells within the CD4^+^ T-cell subset of mice infected with *M. tuberculosis*, compared to the untreated controls (Figure 6B). However, immunization with BCG or treatment with the control IgG or anti-CD25 mAb did not modify the percentages of CD45RB^−^ CD27^−^ cells within the lung CD4^+^ T subset of *M. tuberculosis*-infected mice. Moreover, the frequencies of IFN-γ-producing CD4^+^ T cells from spleen or lungs, in response to stimulation with PPD or immunodominant epitopes of TB10.4 or Ag85A, were comparable in BCG-immunized and *M. tuberculosis*-infected mice that were injected with PBS, the control IgG or anti-CD25 mAb (data not shown). No difference was observed in the TB10.3/4:20–28-specific CD8^+^ T cell responses in these experimental groups (data not shown). Taken together, our results show that, although the attenuation of Treg function increases the anti-mycobacterial Th1 responses subsequent to BCG immunization and at the time of the challenge and at the chronic phase of the infection, the T-cell responses are comparable in all these experimental groups, presumably due to the sustained level of mycobacterial load and the consequent continuous T-cell triggering.

### Anti-CD25 mAb treatment improves moderately but significantly the protective effect of BCG vaccination against infection with *M. tuberculosis*

**Figure 7:**
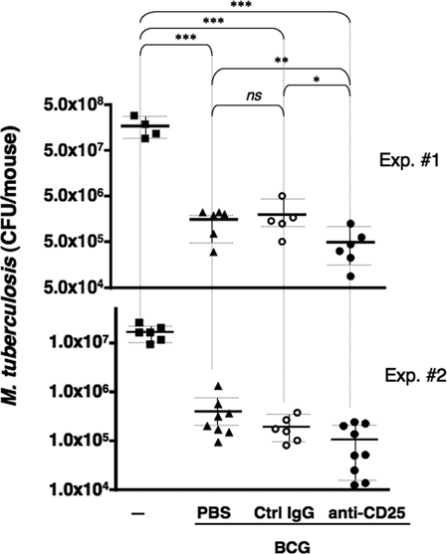
Effect of the attenuation of Treg on the protective capacity of BCG. Evaluation of the protective capacity of BCG in mice treated with anti-CD25 mAb, and challenged with *M. tuberculosis*, performed as shown in Figure 6A. At one month post-challenge, mycobacterial load in the lungs of mice was determined by CFU counting. The number of CFU/lungs at day 1 post-challenge was between 100 and 200 in the experiment 1, and between 10 and 100 in the experiment 2. Horizontal bars and the vertical bars indicate the mean values and SD, respectively. Two independents experiments were performed and data from both of them were taken in account in statistical analyses performed by use of StatXact program and Permutation test. Ns = not significant, *, ** or *** = statistically significant with two-sided *p*-value <0.0420, 0.0046 or 0.0001, respectively.

It has been already shown that anti-CD25 mAb (PC61) treatment has no consequence on the control of mycobacterial growth in the lungs, neither at early, nor at late time points after primary *M. tuberculosis* infection [15]. We thus focused on the effect of anti-CD25 mAb treatment on the control of *M. tuberculosis* infection subsequent to BCG immunization. We determined the mycobacterial load in the lungs of mice either non-immunized or immunized with BCG and treated with PBS, control IgG or anti-CD25 mAb, and challenged with *M. tuberculosis*, as detailed in Figure 6A. BCG immunization decreased the mycobacterial load by 2 log_10_ (Figure 7). Anti-CD25 mAb treatment, concomitant to BCG immunization, reproducibly led to a moderate, albeit statistically significant, decrease of mycobacterial load at the site of infection, i.e., the lung parenchyma (Figure 7, Experiments 1 and 2). In the experiment 1, the Mean±SD of the lung *M. tuberculosis* load in BCG-immunized mice, injected with the control IgG or anti-CD25 mAb was, respectively (1.99±1.7)×10^6^ and (0.45±0.40)×10^6^, which means 4.4 fold less in the latter group. In the experiment 2, the mycobacterial load in BCG-immunized mice, injected with the control IgG or anti-CD25 mAb was, respectively (2.0±1.0)×10^5^ and (1.0±0.9)×10^5^ which means 2 fold less in the latter. Importantly, 5 out of 9 individuals in the last group displayed less than 1×10^5^ CFU/lungs. StatXact program and permutation test (two-sided *p*-value) applied on the results obtained from the experiments 1 and 2 showed that the differences between the anti-CD25 mAb-treated mice and non-Ig-treated or the control Ig-treated mice were statistically significant (*p*<0.0420).

It is noteworthy that based on our long experience, in BCG-immunized mice, the intensity of T-cell responses is of large variability among the individual of the same experimental group. In contrast, such variability was not observed in mice immunized by a single mycobacterial protein (TB10.4 or ESAT-6) in adjuvant. Plausibly, the variability observed in mycobacterial load within the BCG-immunized groups reflects the variability in cell-mediated immune responses in different BCG-immunized individuals.

It is noteworthy that like C57BL/6 mice, BALB/c mice of specific pathogen free status, neither die nor display morbidity, even at 9 months following low dose H37Rv aerosol challenge. Therefore, a possible benefic effect of Treg attenuation during BCG immunization on *M. tuberculosis*–induced host mortality cannot be evaluated in mouse experimental model. Histopathological analyses showed that the anti-CD25 treatment in mice immunized with BCG and then infected with *M. tuberculosis* did not lead to enhanced inflammation or exacerbated lung tissue damage (data not shown). This indicates that dampening the anti-inflammatory Treg subset by anti-CD25 mAb treatment did not increase the inflammation due to mycobacterial infection. These results show that the intervention of Treg, following BCG immunization, contributes partly in limitation of the efficiency of this vaccination in anti-mycobacterial protection.

## Discussion

In the present study, we examined whether a recruitment/expansion of Treg, concomitant to the induction of anti-mycobacterial T-cell responses, could limit the efficiency of BCG vaccine against infection with *M. tuberculosis* in mice. We first observed that, during the four weeks following s.c. immunization with BCG, despite the fact that the percentages of CD25^+^ FoxP3^+^ cells within the CD4^+^ subset remains constant, the total number of these cells increases proportionally to that of the other cells in the inguinal draining lymph nodes, but not in the spleen. The same holds true for the total number of Treg in the lung parenchyma of mice infected with *M. tuberculosis* via the aerosol route. These observations are in accordance with the data published by Quinn *et al.* in which mice, infected with 1×10^4^ CFU of BCG by nasal route or with ≈100 CFU of *M. tuberculosis* by aerosol route, displayed increased size of the Treg compartment, proportional to that of the other lymphocyte subsets [15]. As mentioned in the Results, the increase of the Treg population in these cases may be due to their recruitment, local proliferation/retention or conversion from other CD4^+^ T cells. It is noteworthy that the attenuated BCG, inducer of weak infiltration and inflammation, or virulent and highly persistent *M. tuberculosis*, both induce recruitment/expansion of Treg, while one should expect that virulent mycobacteria would elicit more efficient induction of Treg to avoid tissue injury. This may suggest that induction of Treg is not a consequence of inflammation.

In this study, our approach to evaluate the effect of Treg on anti-mycobacterial adaptive immunity and protection was based on the use of anti-CD25 mAb (PC61) treatment. This strategy has been shown to successfully attenuate *in vivo* Treg functions, without total depletion of this cell subset [19]–[21], in large numbers of various experimental models, including those studying T-cell responses to immunizations, to infections with virus, bacteria and parasites, as well as in anti-tumor immune responses [19], [22]–[24]. In most cases, the T-cell effector functions increase, suggesting that the effect of PC61 treatment is more pronounced on Treg cells — with great majority expressing high levels of CD25 — than on the action of recently activated CD4^+^ T-cell effectors — which transiently express intermediary levels of CD25. Accordingly, the fact that the PC61 treatment does not totally deplete the CD25^+^ T-cell population and that IL2 is not the major or the only one cytokine involved in the activation of CD4^+^ T-cell effectors may explain how PC61 treatment allows attenuation of Treg rather than inhibition of effector functions. Furthermore, the effect of PC61 treatment is relatively persistent, i.e., 12–15 days post-injection [19]–21, making this treatment adaptable to the long experiments of anti-tuberculosis vaccination. At last, long-lasting total depletion of Treg leads to lymphoproliferative disorders [34], [35], which can interfere with the adaptive responses mounted against *M. tuberculosis*.

In mice immunized with BCG and treated by anti-CD25 mAb (PC61), the frequency of IFN-γ producing CD4^+^ T cells specific to the immunodominant epitopes of TB10.3 or TB10.4 immunogens increased significantly. Attenuation of Treg subset by PC61 treatment increased the intensity of IFN-γ responses to all immunodominant and subdominant regions of TB10.4 antigen. Barely immunogenic regions in IgG control-treated, BCG immunized mice, become inducers of easily detectable IFN-γ responses in their PC61-treated counterparts. No new immunogenic regions were revealed under the conditions of Treg attenuation, showing that the repertoire of T cells specific to the different epitopes of TB10.4 is not modified quantitatively. Hence, increase in T-cell responses to subdominant regions may have a direct positive impact on anti-mycobacterial immune-protection, as adaptive immune system becomes able to sense these epitopes more sensitively. Note that TB10.3 and TB10.4 belong to the highly immunogenic ESAT-6 protein family [25] and that TB10.4 is a powerful protective immunogen against *M. tuberculosis* infection [26], [27]. Only a weak and not significant, effect of anti-CD25 mAb treatment was detected on the intensity of CD8^+^ T-cell responses specific to TB10.3 or TB10.4 immunogens, as judged by determination of percentages of such cells by the use of a pentamer of H-2K^d^ restricting element combined with TB10.3/4:20–28 peptide [30], [31].

We then studied the effect of anti-CD25 treatment on the control of the growth of virulent *M. tuberculosis* H37Rv in mice immunized with BCG. In accordance with previous reports [19]–[21], we observed that after injection of anti-CD25 mAb (PC61), the percentage of CD25^+^ FoxP3^+^ cells within the CD4^+^ subset remains decreased, as long as 2 weeks post injection, while at 4 weeks after injection, the Treg subset was totally reconstituted. Based on these data, we performed the anti-CD25 treatment regimen, shown in Figure 6A, to obtain an attenuated Treg compartment: (i) at the time of BCG injection, when anti-mycobacterial T-cell responses are initiated, (ii) during the month following this immunization, when anti-mycobacterial T cells expand, (iii) at the time of *M. tuberculosis* challenge, when the induced anti-mycobacterial T cells encounter the first infected cells, and (iv) at the beginning of the virulence phase of the infection, when the action of such cells controls, at least partially, the growth of mycobacteria. Therefore, in this study, although at 1 month after the challenge, i.e., at day 34 after the last anti-CD25 mAb injection, the Treg subset has been entirely recovered, the phases of initiation, expansion and effector function of anti-mycobacterial T cells occurred under effective attenuation of the Treg compartment. At 1 month post-challenge, when mice possessed substantial mycobacterial load, Th1 responses at the site of infection and in the spleen were comparable in all *M. tuberculosis*-challenged mice, independently of BCG immunization or anti-CD25 mAb treatment. Our most important finding is that the attenuation of Treg subset, by anti-CD25 mAb treatment, concomitant to BCG immunization, moderately but significantly improves the protective efficiency of this vaccine against infection with virulent *M. tuberculosis*. Indeed, BCG-immunized, anti-CD25 treated mice, challenged with virulent H37Rv by aerosol route, displayed a significantly reduced mycobacterial load at the site of infection, compared to their counterparts immunized with BCG alone or immunized with BCG and treated with a control Ig isotype.

Three different strategies have been already used to address the role of Treg in mycobacterial infection. (i) Quinn *et al.* showed that anti-CD25 mAb treatment during the early primary infection of mice with mycobacteria, given by nasal route, increased production of Th1 cytokines but did not reduce the mycobacterial load in the lungs [15]. (ii) Kursar et al. showed that, co-transfer of CD4^+^ CD25^+^ T cells with CD4^+^ CD25^−^ T cells, at a 1∶2 ratio, i.e., markedly higher than the physiological ratio, into *RAG1%* C57BL/6 mice, drastically decreased the capacity of CD4^+^ CD25^−^ T cells to control *M. tuberculosis* load in spleen and lungs. Interestingly, the protective effect of CD4^+^ CD25^−^ T cells in this model was not correlated with any increased IFN-γ or TNF-α responses [16]. (iii) More recently, Scott-Browne et al. set up a mixed chimera C57BL/6 mouse model, reconstituted with bone-marrow cells from Thy1.1 *FoxP3^+/+^* and Thy1.2 *FoxP3%* mice [17]. In these chimeras, infection with *M. tuberculosis* by aerosol route was better controlled when FoxP3^+^ T cells were depleted by anti-Thy-1.1 mAb treatment. Importantly, in this model, the total depletion of Treg compartment induced a substantial polyclonal stimulation of T-cell compartment but did not increase Th1 responses specific to mycobacterial antigens. Therefore, in that study, it was not clear whether the cause of the improved control of *M. tuberculosis* growth was the inhibition of the effect of Treg on specific effector T cells, or a general bystander immuno-activation effect [17]. These three experimental models are very different in their detailed characteristics, yet in all of them, the mycobacterial infection was given into non-vaccinated individuals and therefore it was not evidenced whether prophylactic anti-mycobacterial immunization with BCG gives rise to induction of Treg, able to influence the outcome of the subsequent control of *M. tuberculosis* infection. Our work directly addresses this question in the context of a standard anti-tuberculosis vaccine assay and showed that the efficient attenuation of Treg by PC61 mAb treatment, concomitant to BCG immunization, has a statistically significant, albeit minor, effect on the fine tuning of T-cell immunity controlling *M. tuberculosis* infection.

During the preparation of this manuscript, a first analysis of anti-mycobacterial T-cell immunity and protection by BCG immunization in Treg-attenuation conditions, prior to *M. tuberculosis* challenge, has been published by Quinn *et al* [36]. This team observed that functional attenuation of Treg by PC61 mAb treatment during BCG vaccination increases Th1 responses to Ag85A or to culture filtrate proteins of *M. tuberculosis* but not protection against virulent *M. bovis* or *M. tuberculosis*. Compared to this work performed on C57BL/6 mice [36], our study concerns the effect of Treg in BALB/c mice immunized by BCG, in which we were able to study both CD4^+^ and CD8^+^ T-cell responses to TB10.3 and TB10.4 strong/protective immunogens. Moreover, we characterized the Th1 responses to all the immunodominant and subdominant regions of the TB10.4 antigen. By including high numbers of mice/group and by repeating the protection experiments, our work provides evidence that Treg influence the immune control of *M. tuberculosis* infection.

Several major differences between our experimental conditions and those of the work published by Quinn *et al.* [36] can explain the different results obtained in these two investigations of the effect of Treg on anti-mycobacterial protective capacity of BCG. Indeed, both the pathogen and the host used in these two studies are different. First *M. tuberculosis* Erdman strain has been used in the study by Quinn *et al.* [36] versus *M. tuberculosis* H37Rv strain in the present study. As previously described [37], *M. tuberculosis* Erdman is less virulent than H37Rv in mice and therefore the characteristics of mycobacterial infection are not comparable. Moreover, C57BL/6 mice have been used by Quinn et al *versus* BALB/c mice in the present study. Importantly C57BL/6 and BALB/c mice are genetically distant and therefore the characteristics of mycobacterial infection, i.e., repertoire of T-cell responses, immunodominance of mycobacterial antigens and H-2 restriction are distinct in these two strains. Moreover, BALB/c strain is well known to develop more readily Th2 responses while C57BL/6 mice are prototype of Th1-proned mice. Most importantly, the frequency of Treg has been suggested to be modulated by genetic background [38]. Indeed, this study shows that the frequency of CD4^+^CD25^+^ T cells in the thymus and peripheral lymphoid organs of BALB/c mice is higher than in C57BL/6 mice. Notably, CD4^+^ responder T cells of BALB/c mice show greater susceptibility to suppression of by Treg compare to C57BL/6 mice [38]. Considering all these notable differences, one cannot exclude that the effect of Treg on protective responses can also be quantitatively and qualitatively different in BALB/c and C57BL/6 genetic backgrounds. It is possible that BALB/c strain is a more favorable animal model to detect the effect of Treg in Th1-controlled infectious diseases like tuberculosis. Interestingly, the pioneering work of Y. Belkaid *et al* describing the role of Treg in the control of Leishmmania infection was done in BALB/c mice [13].

Based on our observations, during rational design of improved BCG, it should be considered that, although the action of Treg does not represent the major cause of the limited efficiency of BCG, the impact of this regulatory cell population on the subsequent control of *M. tuberculosis* growth is significant and measurable. In contrast to the anti-CD25 mAb treatment, with mild autoimmune manifestations, germline FoxP3 mutations or general depletion of Treg performed in conditional FoxP3 mutants leads to lymphadenopathy, extensive T-cell activation and severe autoimmune lymphoproliferative disease in neonates and adults [35], [39]. Therefore, it would be of particular interest to evaluate the control of *M. tuberculosis*, after BCG immunization, in an experimental model closer to physiological conditions, for instance under the action of TGF-β — which favors Treg development [39] — or under the combined actions of TGF-β, IL-6 and IL-21, detrimental to Treg development, yet favoring the development of pro-inflammatory Th17 [40]. It has been suggested that the latter can be involved in the recruitment of Th1 cells to the site of *M. tuberculosis* infection [41], even though a direct role of IL-17 in the control of *M. tuberculosis* growth remains to be confirmed [42].

## Materials and Methods

### Mice and Immunization

Female BALB/c mice were purchased from Charles Rivers (Arbresle, France). Six—10-week-old mice received s.c. 1×10^6^ CFU of BCG per mouse. Animal studies were approved by the Institut Pasteur Safety Committee in accordance with French and European guidelines.

### Mycobacteria

*M. bovis* BCG vaccine (Pasteur 1173P2) was kindly provided by G. Marchal (Institut Pasteur, Paris). *M. tuberculosis* (H37Rv), was grown at 37°C in Middlebrook 7H9 medium, complemented with albumine, dextrose and catalase (ADC, Difco, Becton Dickinson, Le Pont-de-Claix, France) and containing 0.05% Tween 80. All experiments done with pathogenic *M. tuberculosis* were performed in a P3 laboratory in accordance with the hygiene and security recommendations of Institut Pasteur.

### Peptides

TB10.3/4:20–28 [30], TB10.3:74–88 [43], TB10.4:74–88 [26], Ag85A:101–120 [44] and PV1:103–116, derived from VP1 protein of type I poliovirus [45], used as a negative control, were all synthetized by NeoMPS (Strasbourg, France).

### Anti-CD25 *in vivo* treatment

Mice were injected i.p. with 0.5 or 1 mg of rat IgG_1_ anti-mouse CD25 mAb (PC61) or of a control rat IgG_1_ (CRL-1912). These Ig were prepared from ascitic fluids precipitated with 50% (NH_4_)_2_SO_4_ at 4°C, in endotoxin-free conditions. Absence of endotoxins in the Ig preparations was then checked by use of “Limulus Amebocytes Lysate” kit (Cambrex, Emerainville, France), with a detection limit of 0.01 IU/ml.

### CD4^+^ T-cell assays

Anti-mycobacterial T-cell responses of immunized mice were measured by IFN-γ-specific ELISPOT, as previously described [46]. Briefly, at day 21 after BCG immunization, serial dilutions of splenocytes from individual mice were cultured for 36 hours in synthetic HL-1 medium (BioWhittaker, Walkersville, MD) complemented with 2 mM L-glutamax, 5×10^−5^ M ß-mercapto-ethanol, 100 IU penicillin/ml and 100 µg streptomycin/ml, in the presence of 10 µg/ml of TB10.3:74–88, TB10.4:74–88 or a negative control peptide, on anti-IFN-γ mAb (R4-6A2)-coated 96-well Multiscreen plates (Millipore, Saint-Quentin-en-Yvelines, France). Biotinylated anti-IFN-γ mAb (XMG1.2), alkaline phosphatase-conjugated streptavidin (PharMingen, Le Pont de Claix, France) and 5-Bromo-5-Chloro-3-Indolyl Phosphate/Nitro“Blue” Tetrazolium (Sigma), as phosphatase substrate, were used to reveal the spots, automatically counted by use of a Bioreader-3000 Pro (BIO-SYS, Karben, Germany). IFN-γ responses in spleen of individual BCG-immunized mice against TB10.4 pepscan, containing 15-mer peptides synthesized in a single amino acid step (Mimotopes, Clayton Victoria, Australia), were assessed as previously described [26]. Briefly, splenoyctes from individual mice were stimulated *in vitro* with 20 µg/ml of each peptide and IFN-γ production was assessed by ELISA after 72 h incubation.

### CD8^+^ T-cell assays

Three weeks after immunization with BCG, splenocytes from mice were stimuled *in vitro* with 10 µg/ml of TB10.3/4:20–28 peptide [30] in RPMI complemented with 2 mM L-glutamax, 5×10^−5^ M ß-mercapto-ethanol, 100 IU penicillin/ml and 100 µg streptomycin/ml and 10% FCS. At day 6, detection of CD8^+^ T lymphocytes specific to TB10.3/4:20–28 epitope was performed by cytofluorometry, by use of a PE-conjugated pentamer of H-2K^d^, complexed with TB10.3/4:20–28 peptide [31] (Proimmune, Oxford, UK). We established that in this pentamer assay, *in vitro* stimulation with TB10.3/4:20–28 peptide did not modify the frequency of the responding mice, compared to *ex vivo* analysis. Indeed, after *in vitro* stimulation, the percentages of the pentamer^+^ cells increase in the same proportion in different individuals and with respect to the initial percentage of the pentamer^+^ T cells detected *ex vivo*. Therefore, following *in vitro* stimulation, the sensitivity of this assay is improved without biasing the frequency of the responding mice or the relative importance of CD8^+^ T-cell responses in different mice.

### Preparation of lymphocytes from lung parenchyma

Lungs were perfused with type IV collagenase (400 U/ml) and DNase I (Roche, Mannheim, Germany). After 45 min incubation at 37°C, 5% CO_2_, lungs were homogenized and passed through nylon filters with 100 µm-diameter pores (Cell Strainer, Becton, Dickinson, Falcon). Cells were then enriched in lymphocytes on a Ficoll gradient (Lympholyte M, Cedarlane Laboratories, Ontario Canada) before cytofluorometric analyses.

### Cytofluorometry

FITC-conjugated anti-CD8 (53–6.7), allophycocyanin-conjugated anti-CD44 (IM7), PE-conjugated anti-CD27 (LG.3A40) and FITC-conjugated anti-CD45RB (16A) mAbs were all purchased from PharMingen. Treg were detected by surface staining with FITC-conjugated anti-CD4 (RM4-5) and allophycocyanin-conjugated anti-CD25 (PC61) mAbs, followed by fixation and permeabilization by use of a mouse Treg staining kit (eBioscience, San Diego, USA) and further intracellular staining with PE-conjugated anti-FoxP3 (FJK16S) or a control isotype (eBioscience), in the presence of FcR-blocking anti-CD16/32 mAb (2.4G2). Cells from *M. tuberculosis*-infected mice were fixed by overnight incubation with 4% paraformaldehyde at 4°C prior to their analysis in a FacsCalibur system by use of CellQuest program (Becton Dickinson Immunocytometry Systems, Mountain View, CA, USA).

### Protection assay against infection with *M. tuberculosis*

Mice were challenged, via the aerosol route, with *M. tuberculosis* H37Rv, by use of a home-made nebulizor. Two ml of a suspension containing 5×10^6^ CFU/ml were aerosolized to obtain an inhaled dose of ≈100 CFU/mouse. Infected mice were placed in isolators. One month post-challenge, lungs were homogenized by use of 2.5-mm diameter glass beads and an MM300 organ homogenizer (Qiagen, Courtaboeuf, France). Serial 5-fold dilutions of homogenates were seeded on 7H11 Agar, supplemented with OADC (Difco, Becton Dickinson, Sparks, MD). CFU were counted after 21 days of incubation at 37°C. Two independents experiments were performed and statistical analyses were performed by use of StatXact program and Permutation test, as described previously [47].
